# Psychological Distress Symptoms and Resilience Assets in Adolescents in Residential Care

**DOI:** 10.3390/children8080700

**Published:** 2021-08-13

**Authors:** Ida Lemos, Marta Brás, Mariana Lemos, Cristina Nunes

**Affiliations:** 1 Psychology Research Centre (CIP) & Universidade do Algarve, Campus de Gambelas, 8005-139 Faro, Portugal; mbras@ualg.pt (M.B.); csnunes@ualg.pt (C.N.); 2 Department of Pediatric Cardiology, Hospital de Santa Cruz, Centro Hospitalar de Lisboa Ocidental, 2790-134 Carnaxide, Portugal; marianatimoteolemos@gmail.com

**Keywords:** psychopathology, resilience assets, residential care, institutionalisation, adolescents, brief symptom inventory, HKRAM (version 6.0)

## Abstract

Most studies with institutionalised children and adolescents focus on evaluating the impact of negative life events on emotional development. However, few have investigated the relationship between resilience assets and the teenagers’ psychopathological problems. The purpose of the present study was to investigate differences in psychological distress symptoms and in resilience assets in institutionalised and non-institutionalised adolescents. A total of 266 adolescents aged between 12 and 19 years old took part in the study (60.5% female): 125 lived in residential care and 144 resided with their families. Results found a significant and inverse relation between psychopathology and the perception of individual resilience assets, specifically with self-efficacy and self-awareness in the community sample, and with empathy in the institutionalised sample. Overall, and regardless of the age group, adolescents in residential care tend to perceive themselves as significantly less resilient in perceived self-efficacy and empathy, and they report fewer goals and aspirations for the future. The importance of promoting mental health and resilience assets in adolescents, particularly in those in residential care, is discussed. This can be achieved through early interventions that may prevent emotional suffering and deviant life paths, with transgenerational repercussions.

## 1. Introduction

### 1.1. Psychosocial Adversity, Psychopathological Problems, and Resilience Assets in Children and Adolescents

Resilience is a developmental and multidimensional process, dependent on sociocultural contexts [1,2,3]. The presence of protective resources in the adolescent’s environment, at a community, school, and family level, may contribute to the strengthening of internal resources or resilience skills [4]. This, consequently, prevents negative outcomes, such as psychopathological problems, and enhances psychological adjustment and well-being in youth.

Studies with community sampling, which focus mainly on the impact of family adversity on behavioural and emotional problems, have shown that emotional closeness to, at least, one main caregiver is essential in the promotion of resilience assets in adolescence [5,6,7]. On this subject, Wolff [8] designated socioeconomic deprivation, family conflicts, divorce, and maternal depression as risk factors for childhood psychopathology. Additionally, Aronen and Kurkela [9] conducted a longitudinal study on the influence of risk factors in the first six months of life and early intervention in families with psychosocial risk. Subsequently, 160 of these subjects were reassessed at 14–15 years of age to evaluate the effects of family factors on their social skills. The results of this study suggest a significant relationship between early risk factors, social skills, and academic achievement. This seems to imply that negative social interactions in early childhood are positively related to poor development of social and academic skills in adolescence. Inversely, the presence of external protective resources contributes to a perception of oneself as resilient, preventing psychological problems in the presence of adversity [2,10].

Children and adolescents who suffer abuse or who live under circumstances of intrafamily violence experience severe and acute stress, and therefore they tend to develop post-traumatic stress disorder. This is usually expressed in the form of emotional and behavioural disturbances and difficulties in interpersonal functioning [11,12,13,14,15]. In this regard, decades of research on psychosocial adversity in childhood, and particularly on child abuse, have produced sufficient evidence to conclude that abuse affects a child’s developmental path, with consequences that can continue throughout life [16]. Thus, this creates a vicious cycle, in which the persistence of psychopathological problems and the changes in the patterns of interpersonal interaction lead to a damaging perception of self and others, which results in more negative experiences [14].

### 1.2. Children and Adolescents in Institutional Care

It has often been noted that the family context is the pillar on which human development is based. For instance, at the beginning of the 21st century, Schoon and Parsons [17], based on the National Child Development Study and on the British Cohort Study, studied two samples of 6801 and 2587 children. The authors managed to bring into evidence the relationship between family characteristics and the impact of sociofamilial adversity on psychosocial adaptation and the development of skills. This is likely because families under social and psychological vulnerability fail to respond to parental roles. Additionally, families in psychosocial risk tend to live in more precarious residential areas, suffer from lack of social support, and must deal with insufficient economic resources. They are also more likely to have mental health issues [18]. All these aspects are associated with dysfunctional family functioning and parental stress, which increase the risk of intrafamily violence and child abuse. Evidently, these can lead to the intervention of social services and the removal of the child or adolescent from the family [19]. In Portugal, the main factors that lead to institutionalisation are parental neglect and abandonment, physical and/or psychological abuse, and the existence of high-risk disruptive behaviour [12].

According to the Report on the Transition from Institutional Care to Community-Based Services in 27 EU Member States [20,21], institutionalisation remains the primary form of care provision with regard to raising youth without parental care or in psychosocial danger, in various countries from the European Union. In Portugal, the Child Protection System relies excessively on institutional care for children and under-relies on family-based care (i.e., foster families or adoption) [21,22].

According to the most recent official data reported by the Portuguese Social Security Institute [23], in 2018, 7032 juveniles were institutionalised in Portugal, 70% aged between 12 and 20 years. Residential or institutional care is a social response designed to support young people until the age of 18 years, in a situation that implies the removal of the child or adolescent from danger; in these cases, the Portuguese child protection system has applied measures for promotion and protection, consisting of residential care [24].

### 1.3. Psychopathological Problems and Resilience Assets in Youngsters in Residential Care

Children and adolescents in foster care suffer several life changes (e.g., separation from caregivers, change of school, adapting to an institutional environment) induced by the institutionalisation. These act as stressors and greatly influence their well-being. Although there are differences among institutional environments, children raised in institutions tend to be exposed to an unfavourable child/caregiver ratio. Thus, this creates a lack of psychological investment by custodians and results in restricted child stimulation. This creates caregiver–child interactions that lack warm, sensitive, and appropriate responsive behaviours [25,26,27]. Several studies on the impact of institutionalisation on children indicate an above normal and long-term prevalence of neurological, physical, cognitive, and behavioural problems. These problems seem to persist even if these children are later raised in socioeconomically privileged families [28,29,30]. For instance, children in the Bucharest Early Intervention Project (BEIP) reported negative sequelae of early institutional care on mental health, with girls expressing significant lower psychopathological symptoms [31]. Moreover, a controlled study of children in foster versus institutional care concluded that the foster care group experienced greater rates of growth in height, weight, and body mass index (BMI) when compared with the institutionalised group [32].

When psychosocial difficulties persist into adulthood, they tend to express themselves through impediments in intimate relationships. Studies that have investigated the parental role of these now grown children have found a shortage of resources necessary to deal with the challenges imposed by child-rearing [19,33].

It is no wonder that because these negative experiences in childhood seem to have psychological and psychosocial long-lasting effects, it is of vital importance to consider which factors lead to fewer psychological resources and maladaptation. These resources or resilience assets are important indicators of global mental health and can provide valuable information about the necessity to intervene in these developmental dimensions. Therefore, the assets or coping mechanisms that an individual may have developed to deal with adversity must be closely studied.

As stated above, individuals in residential care are more vulnerable to psychological hazard because “when we put too many burdens on a kid’s shoulders, he can’t stand up under the weight” [34] (p. 448). It is then fundamental to identify psychopathological problems and their relationship with resilience assets, and to design early interventions that may prevent emotional suffering and inadequate life paths with transgenerational repercussions.

The aim of the present study was to investigate differences in psychological distress symptoms and in internal resilience assets in institutionalised and non-institutionalised adolescents. Moreover, we analysed differences between boys and girls in psychopathology and resilience characteristics.

## 2. Materials and Methods

### 2.1. Design

This is a quantitative, comparative study, with a cross-sectional and descriptive correlational design.

### 2.2. Sample

A total of 266 adolescents aged 12–19 years (*M* = 14.98; *SD* = 1.92) participated in the study (60.5% were girls). A sample of 125 adolescents lived in residential care, and 141 were residing with their families (general population or community sample). The sample-descriptive characteristics according to age, sex, school grade, and parents’ occupational status as well as time and age of institutionalisation (sample of adolescents in residential care) are presented in Table 1.

### 2.3. Measures

#### 2.3.1. Personal Data Questionnaire and Sociodemographic Characteristics

The questionnaire consisted of a set of items related to sociodemographic data. Two versions of the questionnaire were distributed: one was to be completed by residential staff, aimed at collecting specific data about the personal, familiar, institutional, and school context of institutionalised youths; the second version was designed to collect information about the adolescents living with their respective families and aimed to assess individual variables (age, sex, school grade, and parents’ occupation).

#### 2.3.2. Healthy Kids Resilience Assessment Module (HKRAM—(Version 6.0))

The HKRAM (Version 6.0) was developed by Constantine and Benard [35]. The scale consists of 58 questions, which assess 17 protective factors (external resources) and traits of resilience (internal assets). In the present study, only the internal assets scale was applied. It consists of 18 items, each assessing six main developmental positive outcomes or internal resilience assets: (1) cooperation and communication—flexibility in relationships and the ability to work effectively with others, and to effectively exchange information and ideas and to express feelings and personal needs to others; (2) self-efficacy—belief in one’s own competence (e.g., “There are many things that I do well.”); (3) empathy—understanding and caring about another’s experiences and feelings (e.g., “I try to understand what other people feel and think.”); (4) problem-solving skills—ability to plan, to be resourceful, to think critically and reflectively, and to creatively examine multiple perspectives before making a decision or taking action (e.g., “When I need help I find someone to talk with.”); and (5) self-awareness—knowing and understanding one’s self (e.g., “I understand why I do what I do.”); (6) goals and aspirations—using specific dreams, visions, and plans to focus on the future (e.g., “I plan to go to college or some other school after high school.”). The last item also expresses high expectations for one’s self [4]. These six items are organised in a questionnaire in the form of 4-point Likert subscales. In this scale, the respondent must choose an answer on a continuum among highly disagree, disagree, agree, and highly agree. The total scale achieved excellent internal consistency (*α* = 0.93) in its adaptation to Portuguese [36]. The internal consistency of the internal assets scale for this study was good for both samples of institutionalised youth (*α* = 0.87) and non-institutionalised youth (*α* = 0.84).

#### 2.3.3. Brief Symptom Inventory (BSI)

This inventory is a self-report measure evaluating psychological distress and psychiatric symptoms [37]. This questionnaire includes 53 items about current psychological symptoms (e.g., “nervousness or shakiness inside”; “the idea that someone else can control your thoughts”). Through a Likert scale, each participant classifies how often symptoms have occurred in the last 7 days. The BSI is considered a good indicator of general mental health status, with good temporal stability and good discriminative proprieties for the adult Portuguese population [38]. For the purposes of the present study, only the emotional disturbance summary assessment measure—the global severity index (GSI)—was used. The GSI combines the number of psychopathological symptoms and their intensity. This is justifiable by the fact that it is an index summary that allows for a general assessment of the symptoms presented by the subjects. This and the fact that the GSI is composed of the set of BSI items that have high saturations in the dimensions under evaluation make this a logical tool to assess the study’s population. The GSI is calculated through the sum of scores from all indices and then divided by the total number of responses obtained. The Portuguese study of the psychometric properties of the BSI was conducted in a sample of adolescents in a school context, with satisfactory levels of internal consistency (*α* = 0.84) [39]. The internal consistency of the BSI for this study was good for both samples: institutionalised youth (*α* = 0.97) versus non-institutionalised youth (*α* = 0.95).

### 2.4. Data Collection

The study was conducted in the Algarve, which is the southernmost region of Portugal. In the Algarve, there are seven “Temporary care institutions for young people” (residential care), which have the capacity to accommodate 208 young people, according to the Portuguese social protection system. At the time of data collection, the institutions had 152 institutionalised youth.

First, a formal invitation was sent to all Directors of the Temporary care institutions, and of the seven, five responded affirmatively. These five institutions had 138 institutionalised youth. Later, through the institutions, each of the youngsters was individually invited to participate, after informed consent, which made up a total of 125 institutionalised youngsters (13 refused to participate or dropped out—90.6% of the total number of institutionalised youth). To collect information about the adolescents living with their families, data was gathered from a non-randomised sample from a public school in the Algarve. Authorisation was requested by the public-school board, as well as authorisation from the caregivers. All data collection complied with informed consent policies. To comply with relevant and standard ethical principles, the researcher informed all participants of the objectives and relevance of the study and asked for their consent to participate.

Participants, their guardians (technical staff), and caretakers provided their informed consent to participate in the study. The principle of confidentiality of information was maintained, and the partakers were assured that their names would not be mentioned in any stage of the study. The right to opt out of the study was offered with no restrictions. The adolescents were also reassured that they would not be affected by their statements and that all their remarks would remain confidential.

The set of instruments was applied to adolescents, on paper, with the presence of one of the researchers, in a single session, and it was self-administered. The response time was, on average, 40 min. This took place in a classroom context for the participants from the general population sample and in a private room in the teenagers’ residential homes, from December 2019 to May 2020.

### 2.5. Data Analysis

The data was analysed using IBM SPSS 24.0 (IBM Corp., Chicago, IL, USA). Statistical assumptions for parametric analyses were checked following Tabachnick and Fidell’s [40] recommendations, with satisfactory results.

Sociodemographic characteristics were examined between both groups. For this purpose, Snedecor’s F test was used to compare quantitative variables and a chi-squared test was performed for qualitative variables.

Group differences for adolescents’ psychological distress symptoms (BSI) were examined by including group as the independent variable (0 = community sample, 1 = institutionalised sample) and controlling for the adolescent’s sex (0 = girl, 1 = boy).

A MANOVA was conducted to assess whether there were differences between the two groups on a linear combination of all the dependent variables together (internal assets scales of HKRAM). Univariate ANOVAs were used to compare groups when the assumptions of normality and homogeneity of variance were validated.

## 3. Results

Almost all subscales of the HKRAM were significantly related to each other (see Table 2). Psychopathological symptoms evaluated by the BSI (GSI) were significantly and inversely related to self-efficacy and self-awareness in the community sample. In the institutionalised sample, the presence of psychopathology was only significantly and inversely related with empathy.

The ANOVA analysis for BSI (GSI) revealed significant differences between community and institutionalised adolescents’ samples, *F* (1266) = 5.03, *p* < 0.03, *η* = 0.02, after adjusting for the sex of the adolescent. Institutionalised adolescents (*M*_com_ = 1.10, *M*_inst_ = 1.29) and girls reported higher BSI values (*M*_girls_ = 1.36, *M*_boys_ = 0.92).

The MANOVA analysis (including the subscales of the HKRAM—internal assets scale) revealed that community and institutionalised adolescents differed on the HKRAM, *F* (6257) = 10.99, *p* < 0.001, although with a moderate effect size, *η*_partial_ = 0.20 (see Table 3).

Subsequent ANOVAs showed that the self-efficacy (*M*_com_ = 3.13, *M*_inst_ = 2.89), empathy (*M*_com_ = 3.32, *M*_inst_ = 3.06), and goals and aspirations (*M*_com_ = 3.55, *M*_inst_ = 3.06) subscales explained these findings, obtaining higher values in the adolescents in the community.

Regarding sex differences, subsequent ANOVAs revealed that the mean score for girls in the community sample was higher for empathy compared to boys (*M*_com_ girls = 3.46, *M*_com_ boys = 3.11, *F* (1140) = 13.98, *p* < 0.000, *η*^2^ = 0.09). In the institutionalised sample, girls scored higher than boys for cooperation and communication (*M*_inst_ girls = 3.10, M_inst_ boys = 2.60, *F* (1124) =17.53, *p* < 0.000, *η*^2^ = 0.12), empathy (*M*_inst_ girls = 3.30, M_inst_ boys = 2.70, *F* (1124) = 25.26, *p* < 0.000, *η^2^* = 0.17), and goals and aspirations (*M*_inst_ girls = 3.22, *M*_inst_ boys =2.81, *F* (1124) = 10.26, *p* < 0.002, *η*^2^ = 0.08).

## 4. Discussion

### 4.1. Relations between Psychological Distress and Resilience Assets

As grounded in previous empirical research [41], institutionalised teenagers experienced significantly more psychopathological problems when compared to those living with family. Moreover, they reported the presence of fewer resilience resources, which constitute a set of internal resources that are protective from involvement in a maladaptive life course. These resources are cooperation and communication with others, perceived self-efficacy to deal with adversity, empathy towards others’ feelings and experiences, and the ability to dream or make plans (goals and aspirations), when compared with adolescents living with family.

The results in the present study indicate an inverse and significant relationship between psychological distress evaluated by BSI and the internal resilience assets, measured by HKRAM in the adolescents, regardless of their rearing situation. More specifically, self-efficacy and self-awareness were significantly related to psychopathological symptoms in the community sample. Additionally, children in family care who reported higher levels of psychopathological symptoms tended to report themselves as less competent (self-efficacy) and as less self-aware. A previous study by Shibue and Kasai [42] with youth found an inverse relationship between a secure attachment style and resilience levels. Moreover, Galatzer-Levy and Bonanno [43] found significant relations between an anxious attachment style and psychological distress in the same population. This suggests that resilience assets and psychopathological problems may have common mechanisms in their genesis, such as family disfunction or poor quality of the parent–child relationship [10].

In the analysis of children under institutional care, empathy was the only internal asset which was significantly and inversely correlated with psychological distress. It is well known that a lack of empathy translates into a higher difficulty in understanding and caring about another’s experiences and feelings. The relationship between resilience and well-being is influenced by factors such as emotional regulation, adaptive coping strategies, and social skills [35]. Because these characteristics were not directly evaluated in this study, we can only hypothesise about their impact on the way the adolescents express the capability of being empathic to others. According to Fonagy and Allisson [44], secure attachment experiences are fundamental to the formation of empathy—that is, the ability to consider another person’s point of view as reliable, generalisable, and relevant to one’s self.

Furthermore, it is reasonable to assume that besides the consequences of the highly traumatic events that led to institutionalisation, these children carry an additional burden—past and present disturbances in the parent–child relationship—and the adjustment to a residential home should have consequences on the teenagers’ self-perceptions, in their goals and aspirations towards the future, and in their global developmental outcomes. Therefore, adolescents with a history of traumatic events must benefit from environments that offer opportunities to develop empathy, a sense of belonging, a feeling of reciprocity, and of unconditional acceptance [45]. If the biological family cannot offer these opportunities, the community through its care institutions and schools must fulfil the role of establishing a secure foundation for healthy emotional development [46].

### 4.2. Sex Differences

As found in previous research [31] overall, girls from both groups reported significantly higher levels of psychological distress. This corroborates a number of studies that have indicated significantly more emotional difficulties in female adolescents, particularly in what concerns internalising problems [15,47,48,49]. Concerning resilience resources, we found that girls from both samples reported higher levels of empathy when compared to boys. Moreover, institutionalised females also reported higher scores in cooperation and communication and in goals and aspirations. These findings suggest that although, as previously stated, empathy has been systematically reported in the literature as a protective psychological resource [4], high levels of empathic feelings towards others may not serve as a buffer mechanism in what concerns emotional suffering. For instance, it has been suggested by Chikovani and colleagues [50] that high levels of empathy may cause emotional stress by excessively sensitising individuals to negative feelings and situations experienced by others. On the other hand, these results can be due to sex role differences, considering the way cultural contexts influence mentalisation and the expression of emotional suffering [51]. Thus, factors linked to social desirability cannot be disregarded. In Western societies, boys are discouraged from expressing their psychological vulnerability, while girls are encouraged to do so [52].

The fact that boys and girls in residential care seem to be more affected in their well-being, when compared to their peers in the community, can be attributed to their previous negative life events. The fact that their developmental pathways are marked by traumatic family events emphasises the need for the residential care system to work on interventions that focus on current emotional links between caregivers and the child. As previously stated [44], the development of new secure relational patterns can protect against traumatic events experienced in the past.

### 4.3. Resilience in Children in Residential Care

Comparing the two groups of this study—institutionalised versus community—and after adjusting for sex, we find that community and institutionalised adolescents differ in their internal resilience assets. Overall, adolescents in residential care tend to perceive themselves as significantly less resilient in perceived self-efficacy and empathy, and they report fewer goals and aspirations (i.e., minor future expectations for oneself).

These results emphasise that a low perception by adolescents of their internal assets is associated with the expression of psychological distress. In line with the conceptualisations of Constantine and colleagues [4] and Olsson and colleagues [2], resilience must be conceived as a developmental and multidimensional process. Thus, the presence of resources in the adolescent’s environment must contribute to the presence of internal resources or satisfactory resilience skills. These assets will increase the adolescent’s capacity to relate to oneself and to others and to better adapt to the environmental challenges.

In our view, the constructivist perspective [3,53] adds on a complementary and dynamic view to our social ecological model. Therefore, according to Ungar [3], resilience is better understood as a dynamic developmental process, shaped by diverse cultural, political, and social contexts. This means that we are likely to find young individuals who perceive themselves as resilient, despite the maladaptive developmental outcomes they may present. In this line of thought, it is important to analyse not only the past experiences but also the narratives of every adolescent; in other words, the presence of negative life events and the meaning attributed to such negative experiences are equally important.

Some limits and weaknesses of our study need to be considered. A limitation of this study is the lack of a qualitative assessment, which implies that the meanings of competence or of individual resilience resources were not assessed. Moreover, although we found that adolescents in residential care perceived themselves as significantly less resilient in self-efficacy, empathy, goals, and achievements, we are aware that the representativeness of our study is less than ideal due to the sample size, a limitation that possibly constrains some of the conclusions that can be drawn. In addition, the lack of information about child–caregiver interactions should be noted, and also about the influence of residential climate on distress and resilience of institutionalised subjects. This may be due to the difficulty of obtaining reliable objective data from institutional staff on the educational climate of individual residential care.

To improve the quality of residential care, a future study should consider examining the impact of the involvement of an adolescent’s biological parents in that individual’s well-being during custody. Finally, it is important to investigate the impact of the institutional climate on the development of children and youth in residential care.

## 5. Conclusions

Resilience is better defined not in terms of the immediately destabilising effects of trauma and other adverse experiences, but rather by focusing on the increase in the individual’s abilities to overcome trauma, moving to a position of health and positive results, in forms that are culturally and phenomenologically significant [3]. Internal resilience assets are built depending on external resources [35], and therefore the utility of this construct is linked to the requirement to assemble the external resources necessary for the healthy development of children and adolescents. It is the authors’ belief that, despite the incremental change over the years in Portuguese child support policies and the efforts of many social work professionals in the field, studies of psychological distress and of resilience assets in children in residential care are still scarce. In the present study, institutionalised adolescents reported lower levels of resilience and higher levels of psychological distress when compared to adolescents living with their families.

In terms of practical implications, the early identification of psychopathological problems and of the adolescent’s self-perception of resilience assets is important to the design of early interventions. These may prevent emotional suffering and inadequate life paths, with transgenerational repercussions. In the long term, alternatives to residential care must focus on the improvement of the Portuguese foster care system, considering foster families as an alternative to residential care.

## Figures and Tables

**Table 1:** Sociodemographic characteristics of the participants (*n* = 266).

	Adolescents in the Community (*n* = 141)	Institutionalised Adolescents (*n* = 125)	*p*-Value
Age (*M* (*SD*))	15.06 (1.90)	14.90 (1.94)	0.489
Months of institutionalisation (*M* (*SD*))	-	35.80 (39.55)	
Sex			0.484
Male	55	50	
Female	86	75	
School grade			0.001
2nd level (5th–6th grades)	0	33	
3rd level (7th–9th grades)	80	71	
High school (10th–12th grades)	61	21	
Father’s occupation			0.175
Retired	6	11	
Unemployed	17	20	
Active	118	94	
Mother’s occupation			0.071
Retired	0	3	
Unemployed	27	32	
Active	114	90	

**Table 2:** Indexes of correlations between psychopathological symptoms (BSI (GSI)) and resilience (HKRAM) and descriptive statistics by group (adolescents in the community and institutionalised adolescents).

	1	2	3	4	5	6	7
1. BSI (GSI)	-	−0.08	−0.24 **	0.11	−0.07	−0.26 **	−0.10
2. Cooperation and communication	0.14	-	0.34 ***	0.46 ***	0.25 **	0.30 ***	0.27 **
3. Self-efficacy	−0.01	0.53 ***	-	0.15	0.28 **	0.55 ***	0.31 ***
4. Empathy	0.32 ***	0.54 ***	0.43 ***	-	0.35 ***	0.21 *	0.29 **
5. Problem solving	0.06	0.37 ***	0.52 ***	0.34 ***	-	0.32 ***	0.37 ***
6. Self-awareness	0.11	0.46 ***	0.59 ***	0.39 ***	0.31 ***	-	0.54 ***
7. Goals and aspirations	0.08	0.48 ***	0.34 ***	0.39 ***	0.18 *	0.37 ***	-
*M*_com_ (*SD*)	1.10 (0.58)	3.06 (0.62)	3.13 (0.62)	3.32 (0.57)	2.99 (0.74)	3.20 (0.71)	3.55 (0.52)
*M*_inst_ (*SD*)	1.29 (0.88)	2.90 (0.70)	2.89 (0.67)	3.06 (0.72)	2.99 (0.75)	3.17 (0.65)	3.06 (0.72)

Note. BSI (GSI)—brief symptom inventory (global severity index); *M*_com_—mean of adolescents in the community; *M*_inst_ —mean of institutionalised adolescents. Community sample scores on upper-right section and institutionalized sample scores on lower-left section. * *p* < 0.05; ** *p* < 0.01; *** *p* < 0.001.

**Table 3:** Differences on HKRAM between groups (*N*_com_ = 140, *N*_inst_ = 125).

	*F*	*η*_partial_^2^
Control variables		
sex	9.54 ***	0.18
Group	10.99 ***	0.20
Cooperation and communication	4.07	
Self-efficacy	8.63 *	
Empathy	12.08 **	
Problem solving	0.00	
Self-awareness	0.22	
Goals and aspirations	44.16 ***	

* *p* < 0.05; ** *p* < 0.01; *** *p* < 0.001.

## Data Availability

Data can be available to consultation when request to the corresponding author and with permission of the participants of the study.
